# Haemophilia and joint disease: pathophysiology, evaluation, and management

**DOI:** 10.15256/joc.2011.1.2

**Published:** 2011-12-27

**Authors:** Karin Knobe, Erik Berntorp

**Affiliations:** ^1^Lund University, Malmö Centre for Thrombosis and Haemostasis, Skåne University Hospital, Malmö, Sweden

**Keywords:** haemarthrosis, haemophilia, haemophilic arthropathy, joint disease

## Abstract

In patients with haemophilia, regular replacement therapy with clotting factor concentrates (prophylaxis) is effective in preventing recurrent bleeding episodes into joints and muscles. However, despite this success, intra-articular and intramuscular bleeding is still a major clinical manifestation of the disease. Bleeding most commonly occurs in the knees, elbows, and ankles, and is often evident from early childhood. The pathogenesis of haemophilic arthropathy is multifactorial, with changes occurring in the synovium, bone, cartilage, and blood vessels. Recurrent joint bleeding causes synovial proliferation and inflammation (haemophilic synovitis) that contribute to end-stage degeneration (haemophilic arthropathy); with pain and limitation of motion severely affecting patients’ quality of life. If joint bleeding is not treated adequately, it tends to recur, resulting in a vicious cycle that must be broken to prevent the development of chronic synovitis and degenerative arthritis. Effective prevention and management of haemophilic arthropathy includes the use of early, aggressive prophylaxis with factor replacement therapies, as well as elective procedures, including restorative physical therapy, analgesia, aspiration, synovectomy, and orthopaedic surgery. Optimal treatment of patients with haemophilia requires a multidisciplinary team comprising a haematologist, physiotherapist, orthopaedic practitioner, rehabilitation physician, occupational therapist, psychologist, social workers, and nurses.

Journal of Comorbidity 2011;1:51–59

## Introduction

Haemophilia is an X-linked heritable coagulopathy with an overall prevalence of approximately 1 in 10,000 individuals [1]. The most common form is factor VIII deficiency, or haemophilia A, which comprises approximately 80% of cases. Factor IX deficiency, or haemophilia B, comprises approximately 20% of cases [2]. Other heritable clotting disorders include von Willebrand disease and various other clotting factor deficiencies, but these are not commonly classified as ‘haemophilia’ [3]. Haemophilia is traditionally classified as ‘mild’, ‘moderate’, or ‘severe’, depending on the degree of clotting factor deficit compared with that found in the general population (Table 1) [4].

Phenotypically, patients with haemophilia are at risk of haemarthrosis (particularly of the knee, ankle, and elbow joints), soft-tissue haematomas, bruising, retroperitoneal bleeding, intracerebral haemorrhage, and post-surgical bleeding [5]. Generally speaking, individuals with ‘severe’ disease display clinical symptoms more frequently, and are considered at higher risk of haemorrhagic events and musculoskeletal problems, although this is not always the case [4]. Treatment for haemophilia is frequently prophylactic (particularly in moderate or severe disease) and aims to reduce the frequency and severity of bleeds. In addition, treatment needs to be given if the patient has, or suspects they have, a bleed. Replacement therapy uses intravenous infusions of the deficit clotting factor to reduce the risk of bleeding, and patients may receive this at regular intervals (‘prophylactic therapy’) or in response to an acute bleeding episode (‘on-demand therapy’) [6]. It should be mentioned that due to cost and lack of specialized care, access to replacement therapy is, to a large extent, limited to developed countries only. In these countries, children now grow up with a relatively good musculoskeletal status, but this is not yet the case for the majority of patients who live in developing countries and resource-limited settings. Other agents that may be used in the treatment of a bleed include antifibrinolytics (used in both haemophilia A and B) and the synthetic vasopressin analogue desmopressin (only in mild haemophilia A) [7]. Patients are advised to avoid drugs that affect platelet function (although they can be used in some cases), and to avoid trauma/contact sports (although low-impact exercise, such as cycling, is recommended to protect joints) [8].

The management of patients with haemophilia is complex as their condition is associated with a large number of comorbidities. Joint problems resulting from recurrent haemarthrosis, such as chronic synovitis and degenerative arthritis, are a major cause of morbidity [9, 10]. Repeated bleeding into joints can result in abnormalities in both bone growth and limb length [5]. Patients are also at increased risk of developing osteoporosis as a result of prolonged periods of immobility and reduced range of joint movement as a result of arthropathies [11]. Renal haemorrhage and haematuria are also cause for concern, and thromboses resulting from the use of antifibrinolytics may cause renal obstruction. Medications used for other comorbidities, such as blood-borne viral infections, are frequently nephrotoxic and hepatotoxic [5, 12]. Many patients with haemophilia, particularly those transfused before the introduction of virus-safe concentrates, were infected with hepatitis B, C, and HIV. These transfusion-acquired infections increase the risk of end-stage liver disease and cirrhosis, which in turn, increase the risk for hepatocellular carcinoma [13]. While a direct link between haemophilia and cancer is contentious, diagnosis and treatment of malignancy are problematic in these patients [14].

Replacement of missing clotting factors represents an effective method of treatment for patients with haemophilia; however, 20–30% of patients with haemophilia A and 5% of patients with haemophilia B develop inhibitory antibodies to factor VIII and factor IX, respectively, which drastically reduce the efficacy of replacement therapy [15]. This represents a serious complication of treatment, which results in poorer prognosis, reduced quality of life, and increased cost of treatment [16].

Over time, complications from recurrent haemarthrosis and soft-tissue haematomas can result in severe arthropathy, joint contractures, and pseudotumours, leading to chronic pain and disability and impairment of health-related quality of life. Arthropathy as a consequence of haemophilia represents the single largest cause of morbidity in patients with haemophilia, and as such, prevention of this is one of the main aims of treatment [5, 17].

Despite these extensive comorbidities, with appropriate treatment, patients with haemophilia can expect a near-normal life expectancy and an excellent health-related quality of life [18–21]. The effectiveness of treatments means that increasingly, patients with haemophilia are experiencing more of the health concerns associated with advancing age in the general population, such as cardiovascular disease, cancer, and declining renal function [19, 20, 22, 23]. Owing to the increasing age and number of older people living with haemophilia, the incidence of comorbidity and consequently high-risk patients is also increasing, and as survival of patients with haemophilia continues to improve, the impact of comorbidity and how best to manage it will become more important [19, 20, 24]. The presence of haemophilia and comorbidity raises important issues with regard to clinical decision-making and treatment decisions, and currently there is very little guidance for physicians to manage chronic comorbidity. This article reviews comorbid joint disease in haemophilia and how to manage it.

## Haemophilia and joint disease: background

Joint disease is a disabling and common complication of severe (and, to a lesser extent, moderate) haemophilia, in which a characteristic chronic arthropathy develops as a result of recurrent bleeding into joints. Individuals with severe haemophilia are more likely to develop joint problems and reduced range of movement (ROM) of joints [25]. Other risk factors for developing ROM limitations include age and increased body mass index [25]. In those with severe disease, higher frequency of bleeds, presence of inhibitors, and recent orthopaedic procedures are also associated with increased likelihood of ROM limitation [25]. Data from the Universal Data Collection (UDC; US national public health surveillance project) showed that patients with severe haemophilia were more at risk of developing a target joint (a joint in which recurrent bleeding has occurred four or more times in the past 6 months) than those with moderate or mild haemophilia (33.1% versus 18.8% and 5%, respectively) [26].

The most commonly affected joints in patients not treated with prophylaxis are the knees (45%), followed by the elbows (30%), ankles (15%), shoulders (3%), and wrists (2%) [8]. Today, at least in patients on prophylaxis, this pattern appears to have changed, and the ankle joint now accounts for the most common site of bleeding [27]. This may be due to the fact that current prophylactic regimens and treatment in the home allow patients to be more active and able to participate in higher impact sports and activities, which could render the ankle the most vulnerable joint [27]. In individuals with severe haemophilia, the first occurrence of haemarthrosis ordinarily occurs by around the age of 2 years [8]. If not treated adequately, these individuals will develop haemophilic arthropathy by the age of 20 years [28]. An acute bleed into a joint results in severe pain as the pressure in the synovial cavity and bone marrow rises, and may lead to avascular osteonecrosis (particularly in the femoral head following a bleed into the hip joint) [5]. Recurrent bleeding leads to chronic synovitis and damage to both cartilage and bone, in addition to the synovial damage [8]. If patients with severe disease do not receive appropriate treatment, they will develop clinical symptoms: pain, swelling, and reduced ROM by early adolescence that will severely affect their health and quality of life [21, 28].

## Haemophilia and joint disease: pathophysiology

The development of haemophilic arthritis occurs in three stages [29]:

1. Acute haemarthrosis
2. Chronic synovitis
3. Degenerative arthritis.

**Figure 1:**
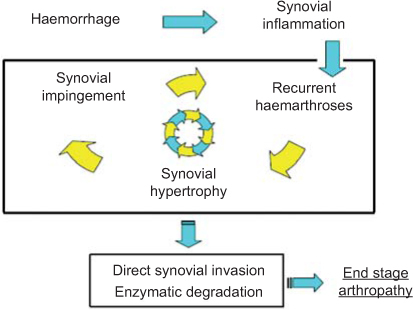
A chronic, self-perpetuating cycle of haemarthrosis–synovitis–haemarthrosis [29]. Reproduced with permission. © World Federation of Hemophilia, 2004.

While the synovium lining a joint has a limited capacity for absorbing blood following an isolated incident of haemarthrosis, recurrent bleeding into the joint results in a level of blood breakdown products that the synovial membrane cannot remove. Iron, a key constituent of haemoglobin found in erythrocytes, is thought to play a major part in inflaming the synovium [29]. The presence of the iron-rich breakdown product haemosiderin is thought to promote the production of pro-inflammatory cytokines such as interleukin (IL)-1, IL-6, and tumour necrosis factor-alpha [30, 31], and the induction of genes that causes cellular proliferation such as *mdm2* [32] that result in the changes seen in synovial tissue in haemophilic synovitis. The synovium becomes increasingly vascular and hypertrophic, and inflammatory cells are recruited to the area in greater numbers. This vascular and hypertrophied tissue is more likely to become impinged between the articular surfaces of the joint, resulting in increased likelihood of further haemarthrosis that creates a vicious cycle of bleeding and inflammation (Figure 1) [29, 33]. Furthermore, the inflammatory mediators released interfere with the normal maintenance of articular cartilage. Damage to the articular cartilage is thought to occur both through direct exposure of the cartilage to blood and through synovium-associated inflammation [34, 35], and it has been shown that the exposure of cartilage to blood, even in the short term, leads to prolonged cartilage damage [33, 34]. The marked inflammation and synovial hypertrophy noted in haemophilic arthropathy bear resemblance to the pathological mechanisms seen in rheumatoid arthritis, while the progressive degeneration of the hyaline cartilage mimics that observed in osteoarthritis. These processes occurring in parallel result in a degenerative arthritis that progresses until the joint is completely destroyed [33, 36].

## Haemophilia and joint disease: evaluation

Clinical evaluation of the joints, gait, motion, muscle tone, functional level of disability, pain and swelling must be performed to assist in the diagnosis of chronic synovitis and to guide treatment decisions. Traditionally, clinical examination and plain film radiography have been used to diagnose haemophilic arthropathy. Radiographs adequately demonstrate advanced bone changes such as epiphyseal overgrowth, joint space narrowing, and osteoporosis, but have poor sensitivity in demonstrating early soft-tissue changes that occur before irreversible cartilage damage [17, 37, 38].

Other imaging methods may be more useful in detecting early soft-tissue changes. Magnetic resonance imaging (MRI) is currently the gold standard for diagnosing haemophilic arthropathy and is particularly useful for identifying soft-tissue changes [39]. MRI can accurately detect synovial hypertrophy and joint effusions, which are common findings at all stages of joint disease. A recent prospective study showed that MRI was more sensitive than radiography in detecting joint abnormalities in boys with severe haemophilia A [17]. However, MRI is often limited owing to high costs, cumbersome use, requirement for sedation in children, and inability to differentiate active versus inactive synovium [38, 40]. Modalities such as contrast ultrasonography may be useful for visualizing synovial changes. The advantages of ultrasonography are that it is simple, inexpensive, convenient, and radiation-free [41–43].

Several scoring systems, including clinical and imaging, have been developed to assess haemophilic joints. The World Federation of Hemophilia (WFH) scoring system, described by Gilbert [44], is based on the clinical evaluation of the six index joints to assess several key parameters of severe haemophilic arthropathy. However, various shortcomings, including lack of established reliability, validity, and sensitivity to smaller changes in patients with less severe joint disease, means that various modifications have been introduced. The current modified clinical system is the Haemophilia Joint Health Score (HJHS) (Table 2) [45]. The Pettersson score [46] and the European MRI scale [47] are imaging techniques that derive the final score from the sum of scores for individually rated features. The Arnold–Hilgartner score [48] and the Denver MRI scale [49] produce radiological scores based on the most severe changes present. The international MRI subgroup has developed a consensus scoring system aimed at facilitating international comparisons between MRI data on haemophilic arthropathy [50].

## Haemophilia and joint disease: management

### Management of bleeding

Optimal management of haemophilic joint disease requires early prevention and treatment of acute joint bleeds before the onset of degenerative disease [8, 17, 29, 51]. Early treatment of joint haemorrhages can be achieved with replacement clotting factor concentrates [5]. The level of clotting factor must be sufficiently high and maintained long enough to stop bleeding and to prevent recurrence [52]. However, despite the success of factor replacement therapy, intra-articular bleeding is still a major clinical manifestation of the disease, particularly in those with severe haemophilia or inhibitors.

Early prophylaxis with factor concentrates in children can prevent not only joint bleeding but also improve joint outcomes, particularly in those with severe haemophilia [17, 53–61]. Prophylaxis is recommended as the first choice of treatment for severe haemophilia by the World Health Organization [57] and the WFH [8] and by many national scientific societies. The Medical and Scientific Advisory Council of the US National Hemophilia Foundation recently recommended prophylaxis as the standard of care for patients of all ages with severe haemophilia [62]. There are currently four models of prophylaxis in haemophilia [see 63]:

1. Primary prophylaxis based on age: continuous, long-term treatment started before the age of 2 years and before any clinically evident joint bleeding (where continuous implies treating up to adulthood for 52 weeks a year, with a minimum of 46 weeks a year)
2. Primary prophylaxis based on first joint bleed: continuous, long-term treatment started before the onset of joint damage, regardless of age
3. Secondary prophylaxis: continuous long-term treatment that does not meet the criteria for primary prophylaxis
4. Short-term prophylaxis: short-term treatment in anticipation of, and to treat, bleeding.

There is a general consensus that prophylaxis with factor concentrates from an early age is the best method for preventing and/or reducing the risk of joint bleeds and arthropathy [17, 59, 60, 61, 63, 64]. The optimal dose, schedule, and timing of prophylaxis remain unclear issues. For maximum benefit, the target trough factor levels should be >1% between dosing [62]. This can usually be achieved by giving 25–50 IU/kg of factor VIII three times a week or every other day [17], or 40–100 IU/kg of factor IX two to three times a week [62]. Dosing is based on the half-life of the factor concentrates, but should be individualized and increased in the case of bleeding [65]. A retrospective comparison of high- and intermediate-dose regimens showed comparable long-term orthopaedic outcomes; the lower dose regimen resulted in a few more bleeds per year, but considerably less factor concentrate consumption [66]. Moreover, a tailored treatment strategy is an option that may require less factor concentrate than ‘traditional’ prophylactic approaches [59].

When to stop primary prophylaxis remains unclear and is poorly studied. Findings from retrospective analyses of prophylaxis in patients with severe haemophilia suggest that some patients, such as those with no, or very few, joint bleeds, may be able to stop prophylaxis in adulthood and switch to on-demand therapy [67, 68]. Results from Denmark and the Netherlands showed that during 4 years of follow-up, one-third of young adult patients with severe haemophilia, who had been receiving prophylaxis during childhood, discontinued prophylaxis in early adulthood, while maintaining a low joint bleed frequency and similar arthropathy to those who continued prophylaxis [68].

For patients with active chronic synovitis and frequently recurring haemarthroses, short treatment courses (6–8 weeks) of secondary prophylaxis with intensive physiotherapy are recommended [8]. In cases where prophylaxis is not feasible or appropriate, on-demand therapy should be given as early as possible at the onset of a bleeding episode [57].

### Adjunctive management

#### Analgesics

Analgesics may be required for the relief of pain due to bleeding into the joint [52]. However, bleeding can be aggravated by analgesics, such as aspiring-containing compounds or other non-steroidal anti-inflammatory drugs [8]. Safer alternatives include paracetamol/acetaminophen and milder opioid analgesics.

#### Anti-inflammatory treatment

When the acute haemarthrosis phase is over, consideration must be given to the synovitis that often develops. Although non-steroidal anti-inflammatory drugs have typically been contraindicated in the bleeding disorder population, each individual should be assessed for the appropriateness of this medication or the more modern variant of cyclo-oxygenase 2 inhibitors. Treatment with intra-articular corticosteroid injection has been described for chronic synovitis [69]. The role of systemic corticosteroids is limited due to their side-effects, but could be considered in specific cases of severe inflammatory reaction refractory to other treatments. The evidence for medical anti-inflammatory treatment in this patient population was recently reviewed by Hermans et al. [70] and considered very low.

#### Rest, ice, compression, and elevation (RICE)

For patients with minor haemarthroses, immobilization may not be required. For other patients, RICE may be useful adjunctive management strategies for pain relief [8]. Ice packs can be applied for 20 minutes, every 4–6 hours, until pain relief. Support to the joint with splints, slings, or pressure bandages can also help to relieve pain. Rest for lower-limb bleeding episodes should include bed rest (1 day), elevation when sitting (3–4 days), avoidance of weight-bearing, and the use of crutches or a wheelchair when ambulating [8]. Immobilization of the painful joint should occur for as short a time as possible and for as long as necessary. However, long-term rest can result in limitation of motion and muscle atrophy [8, 52]. Joint rehabilitation is therefore critical to restore and/or maintain muscle strength and joint ROM.

#### Physiotherapy

Physiotherapy is an important treatment modality to help preserve movement and function to the joints, to reduce swelling and pain, to maintain muscle strength, and to prevent injury. It is important that the physiotherapist is educated about the unique physiology of the haemophilia patient, and makes clinical decisions based on valid research.

Physiotherapy should be initiated as soon as the patient can tolerate it. For subacute haemarthroses, 6–8 weeks of physiotherapy is recommended [71]. Following each factor concentrate injection, the patient should undergo an exercise programme that focuses on active joint mobility, progressive strengthening, gait training, pain management, and self-physiotherapy at home [71]. The physiotherapist will design a specific course of exercises that are tailored to the individual patient. In cases of severe joint damage and surgery, physiotherapy is required for the rehabilitation process. Preventive physiotherapy can be utilized to help strengthen the muscles surrounding the joints, and to improve mobility, flexibility, and balance. A physiotherapist can also provide advice on adopting an appropriate lifestyle and undertaking physiotherapy to prevent and manage bleeds and other musculoskeletal problems [71].

#### Joint aspiration

Joint aspiration is a method for reducing the load of blood after a joint bleed [8]. It can help relieve pain and spasm and speed up rehabilitation [71]. However, there are very limited data on joint aspiration in patients with haemophilia and, except for selected cases, it is generally not recommended in consensus guidelines [70]. Aspiration can be considered in certain circumstances, such as hip haemarthrosis and other major and painful haemarthroses, and should be performed early following a bleeding episode (<12 hours). Joint aspiration may also be considered in patients with acute haemarthrosis who do not respond to factor replacement therapy within 48–72 hours and in cases where pain and swelling outweigh bleeding alone (a septic joint must be ruled out). Aspiration should be performed with adequate factor replacement (factor levels of at least 30–50% for 48–72 hours) [8] and in aseptic conditions to avoid recurrence or septic arthritis [71]. Following aspiration, the joint should remain immobilized for at least 1 hour [8].

#### Surgical treatments

Open surgical procedures are often used for patients with severe joint impairments where conservative therapies have failed. The benefits of surgery must outweigh the potential risks, such as infection, neuropathy, and haemorrhage, particularly in patients with severe haemophilia and/or inhibitors.

#### Synovectomy

For synovitis that is refractory to treatment, but not too hypertrophic and without severe cartilage damage, synovectomy is recommended to prevent both the progression of haemophilic arthropathy and the development of end-stage arthropathy [72]. Synovectomy does not remove the cause of the synovitis, so ongoing management of the underlying disease process influences successful outcome.

The first step to treating synovitis refractory to medical treatment is the use of non-surgical synovectomies (synoviortheses), which involve the percutaneous injection of radioisotopes (yttrium, dysprosium, rhenium, or phosphorus) or chemical agents (rifampicin or oxytetracycline) to generate fibrosis of the hypertrophied synovium [29, 72]. These procedures are minimally invasive, suggested to temporarily preserve ROM, can be performed in the outpatient setting, do not require aggressive physical therapy, require minimal coverage with clotting factor concentrates, can be performed in patients with inhibitors, result in fewer haemorrhagic episodes, and are cheaper than surgical synovectomy [29, 72]. However, complications such as articular cartilage damage, infection, and no relief of symptoms may be associated with the procedure. In addition, two cases of leukaemia have been reported in patients receiving radiosynovectomy using phosphorus 32-sulphur colloid (P32) for haemophilic arthropathy, raising concerns about the safety of this procedure [73, 74]. Thus, the benefits of radiosynovectomy need to be carefully weighed against the potential risks, with careful monitoring for long-term side-effects.

Radiosynovectomy is generally more reliable and quicker at inactivating the synovium than chemical synovectomy [29, 72]. When available, radiosynovectomy is the treatment of choice [29, 72]. Ideally, radiosynovectomy should be performed before irreversible joint destruction has occurred. In cases where radiosynovectomy is not available, chemical synovectomy with rifampicin is recommended. Treatment with up to three consecutive applications of radioisotope at 6-monthly intervals and up to seven applications of chemical agents at weekly intervals are thought to be appropriate before more invasive treatments are considered [72, 75].

Surgical synovectomy may be performed as an open surgical procedure or with the aid of arthroscopy, which avoids the need for large incisions, is associated with less frequent loss of motion, and allows for faster rehabilitation and a more thorough removal of synovial tissue [29, 72]. The procedure may be more effective in younger patients with radiologically less advanced joint arthropathy. However, arthroscopy is considerably more time-consuming than open surgery, and patients still require hospitalization, comprehensive physiotherapy, and large amounts of clotting factor concentrates [29, 72].

#### Joint debridement

Joint debridement is minimally invasive surgery that removes synovitis and loose cartilage from the joint [76]. Arthroscopic joint debridement should be considered in young patients to prevent or delay the requirement for joint arthroplasty. Joint debridement is effective in helping to extend the relatively pain-free, functional life of the joint.

#### Joint arthroplasty

Joint replacement surgery, also known as arthroplasty, is performed when joint pain severely impacts quality of life. The most commonly replaced joints are the knees and hips, with generally good or excellent results, also long term [77].

#### Fusion

This procedure is also known as arthrodesis. Ankle fusion is almost the only fusion procedure carried out today [77]. The surgery involves removal of the painful joint and fusion of the bones. The other joints in the foot will move and thereby provide a close-to-normal gait.

#### Future perspectives

New products with improved pharmacokinetics, allowing for less frequent injections while maintaining therapeutic factor levels, may limit the use of central venous access devices, improve compliance, enable treatment to be initiated earlier, and therefore have the potential to preserve joint function and prevent the onset of arthropathy in non-inhibitor patients. Those with already existing joint disease may instead benefit from the general progress made in many of the medical areas; for example, blood-induced joint damage includes both inflammation-mediated mechanisms as well as cartilage-mediated processes. It has been suggested that IL-10, for example, might be used locally and could therefore modulate joint damage induced by haemarthroses [33].

## Conclusions

Prophylaxis by replacement of the missing factor in patients with haemophilia is the optimal way to prevent the occurrence of haemarthrosis and thereby the onset of arthropathy, provided that it is started early in life. Dosing should be individualized and increased in the case of bleeding. Prevention of bleeding episodes through early treatment will prevent accumulation of blood in the joint and the subsequent inflammation and potential haemophilic arthropathy. Treatment must be maintained until bleeding remission and patients have recovered as much of their ROM and muscular strength as possible. Clinical evaluation of the joints, gait, motion, muscle tone, functional level of disability, pain, and swelling, as well as imaging techniques, must be performed to assist in the diagnosis of chronic synovitis and to guide treatment decisions. The first step to treating synovitis, refractory to medical treatment, is the use of synovectomies, non-surgical or surgical interventions. In many cases, joint deformities have to be treated by open orthopaedic surgery. State-of-the-art treatment of patients with haemophilia requires a multidisciplinary team.

## Figures and Tables

**Table 1:** Classification of haemophilia. Adapted from [4, 7].

Factor level (IU/mL)	Classification	Predisposition to bleeding	Haemarthrosis
>0.05 to 0.40 (>5 to 40% of normal)	Mild	With severe injury, surgery	Rarely
0.01 to 0.05 (1–5% of normal)	Moderate	With slight injury	Sometimes
<0.01 (<1% of normal)	Severe	Spontaneous, with little or no trauma	Very frequently

**Table 2:** Haemophilia Joint Health Score [45].

	Left ankle	Right ankle	Left elbow	Right elbow	Left knee	Right knee	Other
Swelling							
Duration (swelling)							
Muscle atrophy							
Axial alignment							
Crepitus on motion							
Flexion loss							
Instability							
Joint pain							
Strength							
Gait							
Joint total							
Global gait score							
Total score (sum of joint totals + global gait score)							
*Swelling*	*Instability*.						
0 = no swelling	0 = none						
1 = mild	1 = significant pathological joint laxity						
2 = moderate	*Joint pain*						
3 = severe	0 = no pain either through range or at end ROM						
*Duration*	1 = present (observed, grimace, withdrawal or resistance)						
0 = no swelling or >6 months	*Strength* (using Daniels and Worthington’s scale)						
1 = >6 months	Within available ROM						
*Muscle atrophy* 0 = none	0 = holds rest position against gravity with maximum resistance (gr. 5)						
1 = mild	1 = holds test position against gravity with moderate resistance						
2 = severe	(but breaks with maximal resistance) (gr. 4)						
*Axial alignment*	2 = holds test position with minimal resistance (gr. 3+), or holds test position against gravity (gr. 3)						
Measured only at knee and ankle	3 = able to partially complete ROM against gravity (gr. 3−/2+), or able to move through ROM gravity						
0 = within normal limits	eliminated (gr. 2), or through partial ROM gravity eliminated						
2 = outside normal limits	4 = trace (gr. 1) or no muscle contraction (gr. 0)						
*Flexion loss*							
0 = <5							
1 = 5–10							
2 = >20							
*Extension loss*							
0 = <5							
1 = 5–10							
2 = 11–20							
3 = >20							

gr., grade; ROM, range of motion.
